# Toward scientific dissemination of undergraduate thesis in physical therapy programs – a cross-sectional study

**DOI:** 10.1186/s12909-021-03087-8

**Published:** 2022-01-11

**Authors:** Guilherme S. Nunes, Samantha L. Adami, Maitê M. Pellenz, Daniela Rigo, Rafael A. Estivalet, Ane Priscila Diel, Inaihá Laureano Benincá, Alessandro Haupenthal

**Affiliations:** 1 Department of Physical Therapy and Rehabilitation, Federal University of Santa Maria, Av. Roraima, 1000, Santa Maria, RS Postal Code97105-900 Brazil; 2 Department of Health Sciences, Federal University of Santa Catarina, Araranguá, SC Brazil

**Keywords:** Information science, Education, Teacher training, Translational medical research, Evidence-based practice, Scholarly communication

## Abstract

**Background:**

The execution of undergraduate thesis is a period in which students have an opportunity to develop their scientific knowledge. However, many barriers could prevent the learning process. This cross-sectional study aimed to analyze the scientific dissemination of results from undergraduate theses in physical therapy programs and verify the existence of barriers and challenges in the preparation of undergraduate thesis. Second, to investigate whether project characteristics and thesis development barriers were associated with the dissemination of undergraduate thesis results.

**Methods:**

Physical therapists who graduated as of 2015, from 50 different educational institutions, answered an online questionnaire about barriers faced during the execution of undergraduate thesis and about scientific dissemination of their results.

**Results:**

Of 324 participants, 43% (*n* = 138) of participants disseminated their results, and the main form of dissemination was publishing in national journals (18%, *n* = 58). Regarding the barriers, 76% (*n* = 246) of participants reported facing some difficulties, and the main challenge highlighted was the lack of scientific knowledge (28%, *n* = 91). Chances of dissemination were associated with barriers related to scientific understanding and operational factors, such as the type of institution, institutional facilities, and involvement with other projects.

**Conclusion:**

Scientific knowledge seems to be a determining factor for the good development of undergraduate theses. In addition, it is clear the need to stimulate more qualified dissemination that reaches a larger audience. Changes in operational and teaching factors may improve the undergraduate thesis quality. However, the importance of rethinking scientific education within physical therapy programs draws attention.

**Supplementary Information:**

The online version contains supplementary material available at 10.1186/s12909-021-03087-8.

## Background

Undergraduate scientific education is fundamental to stimulate critical thinking, teach how to properly absorb and analyze information, and prepare future health professionals to apply that information in clinical practice [1, 2]. The preparation of an undergraduate thesis (also known as final paper, degree project, bachelor’s thesis, undergraduate dissertation or research project) is perhaps the most direct interaction students have with scientific education [3]. Most undergraduate health programs require students to write an undergraduate thesis [4–6]; therefore, one may infer that undergraduate students contribute a considerable amount of knowledge each year. However, this rarely translates to knowledge dissemination [7–11]. Previous research has demonstrated that the proportion of dissemination in medical and dental undergraduate programs ranges from 17 to 23% [6, 8, 9]. The extent to which undergraduate thesis results are disseminated scientifically in other health programs, such as physical therapy programs, is uncertain.

Undergraduate theses should not be written with the intention of being published in scientific journals. The process is more essential than the outcome. However, scientific dissemination is an important aspect of the learning process, and knowledge of scientific communication is as fundamental as methodological exercise [12, 13]. Qualified publication is also considered to reflect research project success [6, 10, 14], and the small proportion of dissemination may indicate concerns throughout the development of undergraduate theses. The problem is that there is a paucity of research on potential barriers to undergraduate thesis development from the perspective of health students [15]. The study by Frishman [15] described the experience of an education institution in the development of undergraduate theses. From 69 students, 32% of them reported having some difficulty in completing their projects, with the main difficulty being the limited time allotted for the project preparation [15]. Nonetheless, the study included a small number of participants and a high proportion of non-responders (43%) [15]. The identification of potential obstacles and barriers in developing undergraduate theses and disseminating their results could facilitate the implementation of educational strategies aimed at improving scientific skills. Furthermore, challenges in undergraduate thesis development could lead to perspective changes; students may view the undergraduate thesis as an obstacle to obtaining a bachelor’s degree rather than a learning opportunity [16]. Barriers may also lead to the execution of research with low methodological quality, obstructing more qualified scientific dissemination, or there may be little incentive for those results to go beyond the limits of the university center.

### Study goal and the conceptual framework

This cross-sectional study aimed to analyze the scientific dissemination of results from undergraduate theses in physical therapy programs and verify the existence of barriers and challenges in the preparation of undergraduate thesis. Second, to investigate whether project characteristics and thesis development barriers were associated with the dissemination of undergraduate thesis results.

Our conceptual framework was centered on the need to improve scientific dissemination [17] and the lack of scientific education in undergraduate programs [18–21]. Most undergraduate programs have low specialized science teaching workloads in the curricula, therefore students’ development of scientific skills is likely to depend on their participation in laboratory activities or the pursuit of extra-class courses on the subject [19, 22]. Therefore, we can consider the development of undergraduate theses that are crucial for building scientific skills, and a constructive alignment should be applied [23]. In this approach, students interact through their own schemata with teachers’ set up learning environment and are assisted during the project development while being continuously assessed with regard to the intended outcomes. The association of the constructivist methodology with a stimulus to disseminate undergraduate thesis results has the potential to transform students from knowledge consumers to knowledge producers. They transition from the role of a passive receiver of information with a pre-built and embedded meaning from one direction to an individual with a background to think critically and independently, aligning knowledge with reality demands towards new meanings. It is also important to determine what reasons may prevent adequate undergraduate thesis development so that specific educational programs can be established to enhance knowledge dissemination and translation. Based on previous research, we can infer that universities are forgetting a large amount of new knowledge, leading to a waste of resources, unnecessary duplication, and selective publication [24]. Although some solutions have been proposed to promote undergraduate publication [6, 25–27], their effectiveness is debatable, given the low proportion of dissemination [6, 8–11]. The effectiveness of educational programs will likely be improved by understanding the barriers to undergraduate thesis development.

## Methods

### Study design

This was a cross-sectional study reported according to the recommendations of the STROBE (*Strengthening the Reporting of Observational Studies in Epidemiology*) checklist for cross-sectional studies [28].

### Participants

To be included in the study, the participants had to be physical therapists who graduated in Brazilian higher education institutions. Participants who graduated before 2015 were excluded. This criterion was adopted to uniform the sample in terms of time to disseminate the undergraduate thesis results. The sample size was calculated using: *n = (Z*^*2*^ *P (1–P))/d*^*2*^, where *n* is the sample size, *Z* indicates the confidence level, *P* is the expected prevalence, and *d* is the precision [29–31]. Thus, considering *Z* = 1.96, *d* = 5%, and *P* deriving from a pilot study with twenty participants indicating the proportion of people who disseminated their results as 30%, the calculation indicated that at least 323 participants would be necessary to guarantee the reliability parameters. The study was approved by the human research ethics committee of Federal University of Santa Maria (registration number CAAE 41348620.6.0000.5346), and consent was obtained from all participants.

### Procedures

The questionnaire was disseminated through social media posts and emails addressed to alumni by educational institutions. The questionnaire was completed by means of voluntary participation. Data collection was carried out from February to August 2021. Participants were given a link to an online questionnaire (Google Forms tool - Google LLC, Mountain View, CA, USA), that had to be completed just once. For those who answered more than once, only the most recent response was considered.

### Questionnaire

The authors created a preliminary version based on an extensive literature review and records from focus groups [32]. The focus group comprised the authors, recently graduated physical therapists, and undergraduate students working on their undergraduate theses [33]. The questionnaire included closed and open-ended questions with multiple-choice answers, as well as including blank spaces for participants to elaborate the answer. After a panel of experts evaluated the preliminary version for content validity and then, 30 physical therapists and students answered the questionnaire to assess clarity and understanding of the questions. No change was made because the participants did not indicate any clarity or understanding faults.

The final version of the questionnaire (Additional file 1) contained questions on:

(i) *Participant characteristics*: age, graduation year, type of educational institution (public if funded by the government, private if funded by for-profit organizations), and current education level.
(ii) *Undergraduate thesis characteristics*: theme, advisor education level, and involvement of undergraduate thesis projects with other projects.
(iii) *Scientific dissemination*: participants were asked whether they voluntarily disseminated the results of their undergraduate thesis. If so, participants were required to describe the forms of dissemination; otherwise, they had to describe the reasons for the non-dissemination. Participants were advised to consider scientific dissemination as the publication of the results obtained on any means of communication that reached people outside the group participating in the production [12, 34].
(iv) *Experience and perceptions*: participants were required to mention any possible difficulties and barriers in the execution process of undergraduate thesis.

### Data analysis

The answers were analyzed descriptively using frequencies, percentages, and absolute values. To explore the nature of the relationship between results dissemination and the undergraduate thesis characteristics and barriers, three backward stepwise logistic regression analyses were performed. A regression was applied for each of the following dependent variables: a) dissemination of results (yes/no), and publication in scientific journals in which an appropriate *peer review* process is expected – b) publication in international journals (yes/no); c) publication in national journals (yes/no). The following variables were used as potential predictors in all analyses: type of educational institution (public/private) and integration with other projects (yes/no). In addition, the following barriers with frequencies greater than 10% were also included in the analysis as possible predictors, analyzed as present or absent: lack of scientific knowledge, organizational difficulties, lack of time, lack of stimulus to develop a good undergraduate thesis, problems in the student-advisor relationship, lack of adequate facilities at education institutions, and absence of remarkable difficulties. Possible predictors were excluded from the model until the *p*-value for all remaining predictors was smaller than 0.05 [35]. The assumption of multicollinearity was met (tolerance > 0.68) and the inspection of standardized residual values revealed no outliers for dissemination analysis, 10 outliers for international publication analysis (standard residual = 3.72–4.31), and three outliers for national publication analysis (standard residual = 3.36). The outliers were kept in the dataset due to low influence on the models and results. The analyses were conducted using SPSS version 26 (IBM Corp., Armonk, NY, USA).

## Results

We received 415 responses, of which one was from a person who did not consent to participate, 52 from physical therapists who completed their undergraduate program before 2015, and 38 were duplicate responses. Nonresponses were not observed among the valid responses. Hence, 324 participants who graduated from 50 different higher educational institutions were included in the analyses. The characteristics of the participants and their undergraduate theses are presented in Table 1.

**Table 1:** Characteristics of participants and undergraduate theses (*n* = 324)

***Mean age (years)***	27 (SD = 3)
***Mean time since graduation (years)***	3 (SD = 2)
***Current education level (n)***	Undergraduate degree = 87 (27%) Attending a specialization program = 75 (23%) Specialization degree = 62 (19%) Attending a master’s program = 44 (14%) Master’s degree = 36 (11%) Attending a doctorate program = 19 (6%) Doctoral degree = 1 (0%)
***Type of Educational Institution (n)***	Private = 93 (29%) Public = 231 (71%)
***Undergraduate Thesis Theme (n)***	Musculoskeletal = 103 (32%) Respiratory = 57 (18%) Women’s Health = 49 (15%) Neurofunctional = 43 (13%) Sports = 42 (13%) Cardiology = 34 (11%) Pediatrics = 31 (10%) Gerontology = 25 (8%) Primary Care = 19 (6%) Intensive Care = 16 (5%) Electrotherapy = 12 (4%) Manual Therapy = 12 (4%) Oncology = 11 (3%) Experimental = 10 (3%) Others = 39 (12%)
***Education Level of Advisor (n)***	Undergraduate degree = 3 (1%) Specialization degree = 13 (4%) Master’s degree = 56 (17%) Doctoral degree = 205 (63%) Postdoctoral studies = 47 (15%)
***Relationship of Undergraduate Thesis with Other Projects (n)***	Project specific for my thesis = 194 (60%) Project that turned into more than one thesis = 66 (20%) Part of a master’s project = 44 (14%) Part of a project that received funding = 15 (5%) Part of a doctoral project = 11 (3%) Part of a specialization project = 11 (3%) Part of an extension project = 6 (2%) “Umbrella” project = 2 (1%)

Abbreviation: *SD* standard deviation

**Fig. 1:**
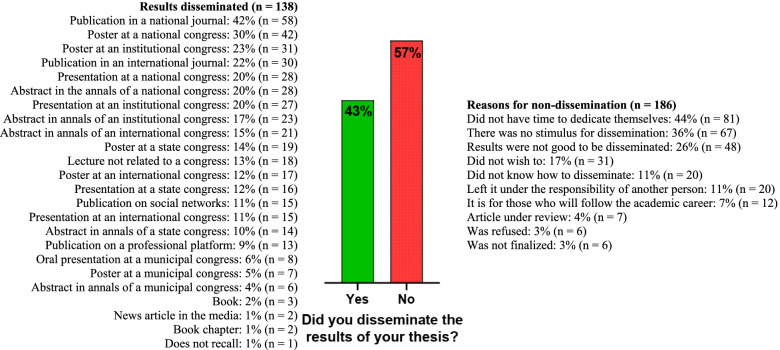
Dissemination of results from undergraduate thesis

In terms of scientific results from undergraduate thesis, 43% (*n* = 138) of participants claimed to have disseminated their results, while 57% (*n* = 186) claimed to have done no dissemination (Fig. 1). The main reasons for the non-dissemination were lack of time (*n* = 81), lack of stimulus to disseminate their results (*n* = 67), and the belief that the results were not good enough to be disseminated (*n* = 48) (Fig. 1).

**Fig. 2:**
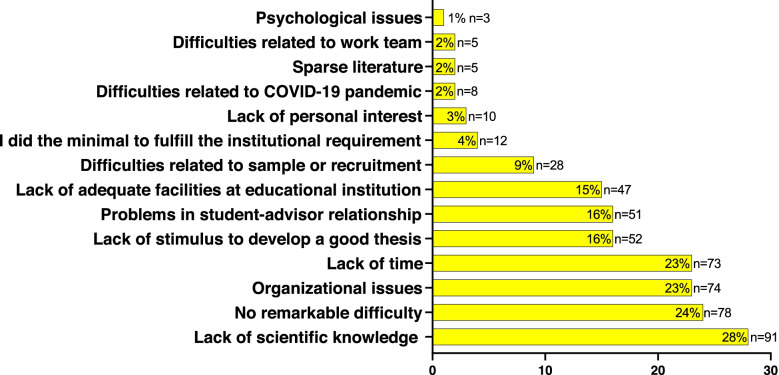
Barriers faced during undergraduate thesis development

Regarding the barriers to develop undergraduate theses, 76% (*n* = 246) of the participants reported facing some difficulties, while 24% (*n* = 78) reported having no remarkable difficulty (Fig. 2). The main challenges highlighted were the lack of scientific knowledge (28%, *n* = 91), organizational issues (23%, *n* = 74), lack of time available to develop the project (23%, *n* = 73), lack of stimulus to develop a good undergraduate thesis (16%, n = 73), problems in the student-advisor relationship (16%, *n* = 51), and inadequate facilities at educational institutions (15%, *n* = 47) (Fig. 2).

The logistic regression results showed that scientific dissemination was associated with attending to undergraduate programs at public institutions (odds ratio [OR] = 3.13, 95% confidence interval [CI] 1.80 to 5.42), not having faced problems in the student-advisor relationship (OR = 3.93, 95% CI 1.81 to 8.52), not having had motivational problems to develop a good undergraduate thesis (OR = 2.27, 95% CI 1.09 to 4.70), and the time for dedication not being an issue (OR = 2.10, 95% CI 1.17 to 3.76) (Table 2). Publishing the results in international journals was associated with attending to programs at public institutions (OR = 7.14, 95% CI 1.63 to 31.36), facing no remarkable difficulty while executing the undergraduate thesis (OR = 5.31, 95% CI 2.12 to 13.28), and the education institution having good facilities (OR = 3.76, 95% CI 1.91 to 11.90) (Table 2). In turn, publishing in national journals was associated with the absence of problems in the student-advisor relationship (OR = 4.92, 95% CI 1.46 to 16.62) and carrying out a project exclusively for the undergraduate thesis (OR = 2.21, 95% CI 1.22 to 4.03) (Table 2).

**Table 2:** Logistic regression results for the analysis of chances to result dissemination

	OR	95% CI	Beta	SE	p
***Dissemination***					
**Absence of student-advisor problems**	3.93	1.81 to 8.52	1.37	0.39	< 0.01
**Public education institution**	3.13	1.80 to 5.42	1.14	0.28	< 0.01
**Absence of motivational problems**	2.27	1.09 to 4.70	0.82	0.37	0.03
**Time for dedicating was not a problem**	2.10	1.17 to 3.76	0.74	0.30	0.01
***International Publication***					
**Public education institution**	7.14	1.63 to 31.36	1.97	0.76	< 0.01
**Absence of remarkable difficulties**	5.31	2.12 to 13.28	1.67	0.47	< 0.01
**Presence of a good structure at institution**	3.76	1.91 to 11.9	1.32	0.59	0.02
***National Publication***^***a***^					
**Absence of student-advisor problems**	4.92	1.46 to 16.62	1.59	0.62	0.01
**Project exclusive for the thesis**	2.21	1.22 to 4.03	0.80	0.31	< 0.01

Abbreviation: *OR* odds ratio, *CI* confidence interval, *SE* standard error

^a^For this analysis, participants with international publications were excluded (*n* = 294)

## Discussion

Although 43% of the sample disseminated their results, only 9% published in international journals and 18% in national journals. Our results revealed that physical therapists experienced relevant barriers throughout their undergraduate thesis execution, which likely contributed to the low rate of qualified scientific dissemination. Relative to the secondary objective of this study, it was possible to identify that having a good student-advisor relationship, attending a public institution, receiving stimulus to build a good project, and having time to conduct the study were all associated with the dissemination of results. Having a good student-advisor relationship and an exclusive project for the undergraduate thesis increases the chances of dissemination for a national article. In turn, being part of a public institution, not facing major difficulties in developing the project, and having an adequate structure to conduct the research were the factors associated with international publication.

Previous studies in medical programs reported that the proportion of dissemination of undergraduate thesis ranges from 11 to 33% [6, 8–11] and master’s thesis results were published by 22 to 30% of the students [36, 37]. A higher proportion of dissemination was observed in physical therapy master’s programs, ranging from 45 to 54% [38, 39]. In terms of general dissemination, our study presented a similar dissemination proportion to previous studies (43%). However, if we consider only international publications, we can see that our percentage is relatively low (9%). Nevertheless, we may conclude that degree project results are poorly disseminated, and their visibility should be improved. Few research, involving health undergraduate students, has been conducted on the challenges to completing an undergraduate thesis [15]. Studies have been conducted on the participation of undergraduate medical and dental students in research activities, and the related barriers are similar to those reported in this study, such as lack of knowledge [40], lack of time [41], and issues in the student-advisor relationship [42]. The literature covers various aspects related to scientific publication [11, 43, 44], but specific factors related to undergraduate thesis have received little attention. The most important factors related to publication seem to be having adequate facilities and receiving grants to conduct research [43, 44].

Our results are limited due to methodological factors. Only physical therapists who graduated in Brazilian institutions were included, which may limit the results to this group. However, this study is a pioneer in investigating the factors that influence the execution of undergraduate theses in physical therapy programs, and other countries may present similar barriers. Another limitation could be the large number of participants who graduated from public universities, which may have influenced the results. In any case, our results may be seem as an appropriate reflection of undergraduate physical therapy programs and their relationship with scientific education. In addition, our analyses included a few participants classified as outliers. We observed that maintaining outliers in the dataset had a minimal impact on the consistence of the regression models.

The primary means of dissemination among the participants was national journals and congresses, limiting the information to a domestic or Portuguese speaking audience. We are not claiming that the focus of scientific dissemination should be publishing in international journals. The existing scientific publication model is neither ideal nor a synonymous with appropriate far-reaching scientific dissemination, and other means of communication are also important and may reach an even wider audience [45]. However, the editorial process of peer-reviewed scientific journals ensures greater reliability of the information because the content is assessed before it is published [46, 47]. When much disinformation is disseminated [48, 49], techniques such peer review bring a higher level of security [50].

Barriers and difficulties are a part of the learning process and execution of any academic work [51], including undergraduate thesis writing, as observed in this study. A finding that draws attention is that over one-fourth of the participants reported a lack of scientific knowledge. This may reflect issues in scientific education throughout programs, and students may be hesitant in executing research projects without strong theoretical foundations [52]. Besides the increase in the workload dedicated to the development of scientific skills, the literature offers further recommendations for improving scientific learning in undergraduate programs [18–21]. For instance, active student engagement in knowledge building appears to be a determining factor [18, 20, 21]. Teachers should shift their focus from teaching specific scientific methodologies to a form of teaching that encourages scientific reasoning [18, 20, 21]. Scientific education does not need to be limited to “scientific bases” or “evidence-based practice” courses. Instead, scientific themes could be incorporated into each course and the activities that compose the curriculum.

By gaining scientific skills to overcome the main identified barrier, other obstacles may be resolved. The ignorance on the foundations of scientific processes may lead to problems in scientific planning, such as the formation of scientific questions that do not correspond to the academic realities of students or institutions. Students who do not perform proper planning may involve themselves in difficult-to-execution projects, which will entail organizational challenges, the need to dedicate more time, and, finally, a sense of discouragement, interfering in the relationships with their advisors. Moreover, motivating students by demonstrating the importance of an undergraduate thesis and making them feel emotionally connected to the work may be fundamental [52]. A point that reinforces the importance of working out the difficulties is that participants who did not face remarkable difficulties were approximately five times more likely to publish their results in international journals.

Operational issues were also related to disseminating results and requiring the attention of teachers and educational institution managers. Institutions with a lack of structure reduce the chances of being published in international journals. However, the most important operational factor seems to be the type of educational institution, as studying at a public institution increased the probability of publishing in an international journal by seven times. This may reflect the Brazilian scientific reality, where public education institutions are the primary research centers, concentrate the majority of graduate programs, and receive considerably more investments for research than private institutions [53, 54], all of which are important factors for scientific production [44, 55]. This suggests that private institutions should reconsider the role of research in their programs, and public investment in scientific production institutions should be raised or maintained to support scientific growth and knowledge generation [56, 57].

Undergraduate thesis development is a form of active learning in which students practice the skills such as writing, thinking and argumentation, as well as clinical skills [2, 58]. Teachers and advisors should abandon a strategy centered on knowledge transmission and regard undergraduate thesis as a project conducted by and for students. Consequently, the process becomes more dynamic, oriented on students, and implementing an adaptive form of learning in accordance with students’ request [59]. In addition, if we approach this process as a means to connect clinical practice with research, we may aid knowledge transfer from universities to everyday professional practice [60]. Accordingly, teachers and advisors must provide the necessary assistance to overcome the barriers identified in this study, thereby improving the learning potential [58].

The present study highlights the vast amount of new knowledge that exist within university walls. Educational institutions’ managers and advisors should encourage the dissemination of undergraduate thesis results by publicizing university activities and collaborating with building stronger literature. Furthermore, the importance of scientific education becomes evident, as this may facilitate the execution of undergraduate theses and, consequently, expand the transmission of new knowledge. Future research could promote various educational programs and methodologies centered on scientific teaching and evaluate their impact on different aspects of undergraduate thesis execution, such as barriers to execution and rate of dissemination. Even though publication of the results does not necessarily reflect what students have learned or developed, we can better understand how different factors influence this output by comparing practices that led to published and unpublished research projects in order to implement educational strategies that enrich students’ and advisors’ experience.

## Conclusion

The results of the undergraduate thesis of physical therapy programs were disseminated by less than half of the participants, with most common method of dissemination being publication in national journals. The majority of participants reported that they faced difficulties in executing their undergraduate thesis, with a lack of scientific knowledge being the main barrier. Public educational institutions, an absence of remarkable difficulties, a friendly relationship between students and advisors, educational institutions with adequate facilities, projects exclusively designed for the thesis, motivated students, and available time to dedicate for project execution were all associated with scientific dissemination of results from the undergraduate thesis of physical therapy programs.

## Supplementary Information

**Additional file 1..**

## Data Availability

The datasets used and/or analyzed during the current study are available from the corresponding author on reasonable request.

## Abbreviations

STROBE: Strengthening the Reporting of Observational Studies in Epidemiology
OR: Odds ratio
CI: Confidence interval
