# Supplementary material for: Toward scientific dissemination of undergraduate thesis in physical therapy programs – a cross-sectional study
Source: BMC Med Educ. 2022 Jan 11;22:32. doi: 10.1186/s12909-021-03087-8 (PMC8753828; doi:10.1186/s12909-021-03087-8)
Supplement: Supplementary file 1 — Additional file 1.. [file 12909_2021_3087_MOESM1_ESM.pdf]

UNDERGRADUATE THESIS – PERCEPTIONS AND BARRIERS

You are invited to answer the below survey that intend to register your experience during the development of your undergraduate thesis which was required to conclude the physical therapy program.

Participant characteristics

- Name:
- Email:
- Age:
- Graduation year:
- Type of educational institution in which you have graduated

( ) Public
( ) Private

- What is your current educational level?

( ) Undergraduate degree
( ) Attending a specialization program
( ) Specialization degree
( ) Attending a master's program
( ) Master's degree
( ) Attending a doctorate program
( ) Doctoral degree

Undergraduate thesis characteristics

- What was the theme of your undergraduate thesis? You can select more than one alternative and/or use the blank space to add other themes.

( ) Aesthetics	( ) Neurofunctional
( ) Cardiology	( ) Oncology
( ) Dermatofunctional	( ) Pediatrics
( ) Electrotherapy	( ) Primary Care
( ) Experimental studies (animals)	( ) Respiratory
( ) Gerontology	( ) Sports
( ) Intensive Care	( ) Women's Health
( ) Manual Therapy	( ) Work health
( ) Musculoskeletal	( ) *blank space:*

- What was the educational level of your advisor?

( ) Undergraduate degree
( ) Specialization degree
( ) Master's degree
( ) Doctoral degree
( ) Postdoctoral studies

- Was your undergraduate thesis involved with other projects? If yes, please, indicate the relationship. You can select more than one alternative and/or use the blank space to add other relationships.

( ) Project specific for my thesis	( ) Part of a postdoctoral project
( ) Project that turned into more than one thesis	( ) Part of an extension project

( ) Part of a specialization project<br>( ) Part of a master's project<br>( ) Part of a doctoral project	( ) Part of a project that received funding<br>( ) "Umbrella" project<br>( ) *blank space :*

Scientific dissemination

• Did you voluntarily disseminate the results of your undergraduate thesis?<br>( ) Yes ( ) No	
• If "yes", please, indicate the places where you have disseminated your results	
( ) Publication in an international journal<br>(*paper written in non-Portuguese language*)<br>( ) Publication in a national journal<br>(*paper written in Portuguese*)<br>( ) Presentation at an international congress<br>( ) Presentation at a national congress<br>( ) Presentation at a state congress<br>( ) Presentation at a municipal congress<br>( ) Presentation at an institutional congress<br>( ) Lecture not related to a congress<br>( ) Poster at an international congress<br>( ) Poster at a national congress<br>( ) Poster at a state congress<br>( ) Poster at a municipal congress	( ) Poster at an institutional congress<br>( ) Abstract in annals of an international congress<br>( ) Abstract in the annals of a national congress<br>( ) Abstract in annals of a state congress<br>( ) Abstract in annals of a municipal congress<br>( ) Abstract in annals of an institutional congress<br>( ) Book<br>( ) Book chapter<br>( ) Publication on social media<br>( ) Publication on a professional platform<br>( ) Publication on blogs<br>( ) News article in the media<br>( ) *blank space:*
• If "no", please, indicate the reasons for the non-dissemination of your results:	
( ) Did not wish to<br>( ) There was no stimulus for dissemination<br>( ) Results were not good to be disseminated<br>( ) Did not know how to disseminate<br>( ) I do not think it is important disseminating my results	( ) Left it under the responsibility of another person<br>( ) Did not have time to dedicate themselves<br>( ) It is for those who will follow the academic career<br>( ) *blank space:*

Experience and perceptions

• Please, indicate difficulties which you faced during the development of your undergraduate thesis. You can select more than one alternative and/or use the blank space to add other difficulties.	
( ) No remarkable difficulty<br>( ) Lack of personal interest<br>( ) Lack of time<br>( ) Lack of scientific knowledge<br>( ) Problems in student-advisor relationship<br>( ) I did the minimal to fulfill the institution requirement	( ) Lack of adequate facilities at educational institution<br>( ) Lack of stimulus to develop a good thesis<br>( ) Organization difficulties<br>( ) *blank space:*
